# Predictors of renal function recovery among patients undergoing renal replacement therapy following orthotopic liver transplantation

**DOI:** 10.1371/journal.pone.0178229

**Published:** 2017-06-02

**Authors:** Maria Claudia Cruz Andreoli, Nádia Karina Guimarães de Souza, Adriano Luiz Ammirati, Thais Nemoto Matsui, Fabiana Dias Carneiro, Ana Claudia Mallet de Souza Ramos, Ilson Jorge Iizuca, Maria Paula Vilela Coelho, Rogério Carballo Afonso, Ben-Hur Ferraz-Neto, Marcio Dias de Almeida, Marcelino Durão, Marcelo Costa Batista, Julio Cesar Monte, Virgílio Gonçalves Pereira, Oscar Pavão dos Santos, Bento Cardoso dos Santos

**Affiliations:** 1 Centro de Diálise Einstein, Hospital Israelita Albert Einstein, Sao Paulo, Brazil; 2 Unidade de Transplante de Fígado, Hospital Israelita Albert Einstein, Sao Paulo, Brazil; University of Toledo, UNITED STATES

## Abstract

Renal dysfunction frequently occurs during the periods preceding and following orthotopic liver transplantation (OLT), and in many cases, renal replacement therapy (RRT) is required. Information regarding the duration of RRT and the rate of kidney function recovery after OLT is crucial for transplant program management. We evaluated a sample of 155 stable patients undergoing post-intensive care hemodialysis (HD) from a patient population of 908 adults who underwent OLT. We investigated the average time to renal function recovery (duration of RRT required) and determined the risk factors for remaining on dialysis > 90 days after OLT. Log-rank tests were used for univariate analysis, and Cox proportional hazards models were used to identify factors associated with the risk of remaining on HD. The results of our analysis showed that of the 155 patients, 28% had pre-OLT diabetes mellitus, 21% had pre-OLT hypertension, and 40% had viral hepatitis. Among the patients, the median MELD (Model for End-Stage Liver Disease) score was 27 (interquartile range [IQR] 22-35). When they were listed for liver transplantation, 32% of the patients had serum creatinine (Scr) levels > 1.5 mg/dL or were on HD, and 50% had serum creatinine (Scr) levels > 1.5 mg/dL or were on HD at the time of OLT. Of the transplanted patients, 25% underwent pre-OLT intermittent HD, and 14% and 41% underwent continuous renal replacement therapy (CRRT) pre-OLT and post-OLT, respectively. At 90 days post-OLT, 118 (76%) patients had been taken off dialysis, and 16 (10%) patients had died while undergoing HD. The median recovery time of these post-OLT patients was 33 (IQR 27–39) days. In the multivariate analysis, fulminant hepatic failure as the cause of liver disease (p<0.001), the absence of pre-OLT hypertension (p = 0.016), a lower intraoperative fresh-frozen plasma (FFP) transfusion volume (p = 0.019) and not undergoing pre-OLT intermittent HD (p = 0.032) were associated with performing RRT for less than 90 days. Therefore, a high proportion of OLT patients showed improved renal function after OLT, and those who were diagnosed with fulminant hepatic failure, had no pre-OLT hypertension, received a lower transfused volume of intraoperative FFP and did not undergo pre-OLT intermittent HD had a higher probability of recovery.

## Introduction

In the MELD (Model for End-Stage Liver Disease) score era of organ allocation, which considers serum levels of creatinine and bilirubin and the international normalized ratio of the prothrombin time, patients are prioritized based on measures of preoperative renal dysfunction. Between 5% and 35% of liver transplant patients require perioperative dialysis for renal replacement therapy (RRT) [1]. Information regarding a patient’s RRT duration requirements and probability of kidney function recovery is crucial for transplant program management.

Patients with liver disease and advanced irreversible renal disease are typically considered for a combined liver and kidney transplant [2, 3]. In contrast, patients with acute kidney injury (AKI) can have improved renal function after orthotopic liver transplantation (OLT) and thus are considered for isolated liver transplantation. AKI patients may remain on RRT post-transplant for various periods of time, and some may never spontaneously recover renal function. These patients will then need a kidney transplant [4].

Isolated liver transplant recipients who remain on RRT during the post-transplant period have lower survival rates than those who undergo subsequent kidney transplantation [5, 6]. However, given the shortage of available organs, it is essential to determine the optimal length of time a physician should wait before deciding that a patient undergoing post-OLT RRT has an irreversible AKI and thus needs kidney transplantation. This approach may avoid unnecessary transplantation in a patient whose native kidney function could eventually improve.

We investigated a group of 155 stable patients undergoing post-intensive care dialysis following OLT to evaluate the average time to renal function recovery (duration of RRT) and factors associated with remaining on dialysis > 90 days after OLT in an urban tertiary medical center in São Paulo, Brazil. We consider renal function recovery the suspension of dialysis, and the average time to renal function recovery (duration of RRT) refers to the interval between the date of OLT and the date of the last hemodialysis session performed.

## Materials and methods

### Study population

In this retrospective study, we evaluated a sample of 155 stable patients undergoing post-intensive care dialysis from a patient population of 908 adults who underwent OLT between June 1, 2005, and December 31, 2011, at Albert Einstein Jewish Hospital, São Paulo, Brazil, an urban tertiary medical center. All the patients required RRT [intermittent hemodialysis (HD)] for presumed AKI during the postoperative period. Combined liver-kidney transplant recipients were excluded from the study due to the possibility of concurrent chronic kidney disease.

For each patient, the following data were collected: age; gender; pre-transplant diabetes mellitus and hypertension status; etiology of liver disease (viral hepatitis, alcoholic cirrhosis, fulminant hepatic failure or other); MELD score; history of hepatocellular carcinoma; pre-OLT continuous renal replacement therapy (CRRT) and pre-OLT intermittent HD; history and volume of intraoperative packed red blood cells, fresh-frozen plasma (FFP), and cryoprecipitate and platelet transfusion; length of intensive care unit (ICU) stay during transplant hospitalization; and duration of pre-OLT RRT. CRRT was performed as continuous venovenous hemodiafiltration (CVVHDF) with the Prismaflex® system (Gambro, Lakewood, CO, USA) in hemodynamically unstable patients in the ICU. The need for early liver retransplantation (re-OLT) or post-OLT CRRT was noted. Additionally, the presence of serum creatinine (Scr) levels > 1.5 mg/dL or the need for RRT at the time of listing for liver transplantation or at the time of transplant was determined. The etiology of renal dysfunction was not assessed in our study due to its frequent inaccuracy. Tacrolimus levels were routinely monitored and were kept within the therapeutic range.

We evaluated the average time to renal function recovery in OLT patients undergoing post-intensive care hemodialysis (HD) and determined the risk factors for remaining on dialysis > 90 days after OLT. Renal function recovery was defined as no longer needing RRT. Patients were censored at the time of recovery of kidney function, death on HD, or at the end of follow-up.

### Statistical analysis

Unadjusted post-transplant survival was estimated using Kaplan-Meier curves and compared using log-rank tests. Multivariate Cox proportional hazards models were used to evaluate factors associated with renal function recovery, and the results are presented as hazard ratios (HRs) and 95% confidence intervals (CIs) for those remaining on HD after 90 days. The data were analyzed using SPSS (Statistical Package for the Social Science) 17.0 for Windows. Statistical significance was set at p < 0.05. Continuous and categorical variables were expressed as medians [quartile 1 (Q1) to quartile 3 (Q3)] and as counts and percentages, respectively.

Approval was obtained from the local ethics committee, and formal informed consent was waived due to the observational nature of the study (Ethics Committee: Hospital Israelita Albert Einstein - Sao Paulo – Brazil; Approval Number: 37516814.3.0000.0071). The clinical and research activities reported here are consistent with the Principles of the Declaration of Istanbul as outlined in the “Declaration of Istanbul on Organ Trafficking and Transplant Tourism”.

## Results

### Baseline characteristics

A total of 155 adult recipients [male: female 99:56; median age 53.1 (45.0 – 60.3) years] were included in our study. Of the patients, 43 (27.7%) had pre-transplant diabetes mellitus, and 32 (20.6%) had pre-transplant hypertension; viral hepatitis was the cause of liver disease for 62 (40.0%). The median MELD score was 27 (22-35). Overall, 47 (31.5%) patients had Scr levels > 1.5 mg/dL or were on HD at the time of listing, and 78 (50.4%) patients had Scr levels > 1.5 mg/dL or were on HD at the time of OLT. Twenty-six patients (16.8%) needed early re-OLT: 20 (77%) for primary nonfunction, 5 (19%) for hepatic artery thrombosis and 1 (4%) for hyperacute rejection. Twenty-eight patients (18.1%) had a history of hepatocellular carcinoma. Of the patients, 21 (13.5%) underwent pre-OLT CRRT, 38 (24.5%) underwent pre-OLT intermittent HD and 63 (40.6%) underwent post-OLT CRRT (undergoing CRRT and intermittent HD were not mutually exclusive). The median intraoperative transfusion volumes were 2 (0-3) U packed red blood cells and 0 (0-5) U FFP; 22 (14.2%) and 47 (30.3%) of the patients received intraoperative cryoprecipitate and platelet transfusions, respectively. The median length of ICU stay during transplant hospitalization was 7 (4-14) days. One hundred nine (70.3%) patients did not undergo pre-OLT RRT, and 37 (23.9%) underwent ≤ 14 days and 9 (5.8%) underwent > 14 days of pre-OLT RRT (Table 1). The median length of pre-OLT RRT was 5 (2-11) days. Table 1 also shows the baseline characteristics of the stable patients undergoing post-OLT RRT by renal function recovery status.

**Table 1:** Baseline characteristics of stable patients undergoing RRT post-OLT according to renal recovery status (N = 155).

Population characteristics		Renal function recovery ≤ 90 days		Total
		No (N = 37)	Yes (N = 118)	
Age (years)		55.1 (48.8 – 61.2)	52.8 (42.5 – 59.4)	53.1 (45.0 – 60.3)
Gender				
	Male	20 (20.2%)	79 (79.8%)	99 (63.9%)
	Female	17 (30.4%)	39 (69.6%)	56 (36.1%)
Pre-OLT diabetes mellitus				
	No	25 (22.3%)	87 (77.7%)	43 (27.7%)
	Yes	12 (27.9%)	31 (72,1%)	112 (72.3%)
Pre-OLT hypertension				
	No	26 (21,1%)	97 (78.9%)	123 (79.4%)
	Yes	11 (34.4%)	21 (65.6%)	32 (20.6%)
Etiology of liver disease				
	Viral hepatitis	17 (27.4%)	45 (72.6%)	62 (40.0%)
	Alcoholic cirrhosis	2 (11.1%)	16 (88.9%)	18 (11.6%)
	Fulminant hepatic failure	1 (5.9%)	16 (94.1%)	17 (11.0%)
	Other	17 (29.3%)	41 (70.7%)	58 (37.4%)
MELD		26 (24 – 36)	28 (20 – 35)	27 (22-35)
Scr level > 1.5 mg/dL or undergoing RRT at the time of listing*				
	Scr level ≤ 1.5 mg/dL	26 (25.5%)	76 (74.5%)	102 (68.5%)
	Scr level > 1.5 mg/dL	9 (22.5%)	31 (77.5%)	40 (26.8%)
	RRT	2 (28.6%)	5 (71.4%)	7 (4.7%)
Scr level > 1.5 mg/dL or undergoing RRT at the time of transplant				
	level ≤ 1.5 mg/dL	14 (18.2%)	63 (81.8%)	77 (49.7%)
	Scr level > 1.5 mg/dL	6 (20%)	24 (80.0%)	30 (19.4%)
	RRT	17 (35.4%)	31 (64.6%)	48 (31.0%)
Early re-OLT				
	No	34 (26.4%)	95 (73.6%)	129 (83.2%)
	Yes	3 (11.5%)	23 (88.5%)	26 (16.8%)
Hepatocellular carcinoma				
	No	30 (23.6%)	97 (76.4%)	127 (81.9%)
	Yes	7 (25.0%)	21 (75.0%)	28 (18.1%)
Pre-OLT CRRT				
	No	31 (23.1%)	103 (76.9%)	134 (86.5%)
	Yes	6 (28.6%)	15 (71.4%)	21 (13.5%)
Pre-OLT intermittent HD				
	No	23 (19.7%)	94 (80.3%)	117 (75.5%)
	Yes	14 (36.8%)	24 (63.2%)	38 (24.5%)
Post-OLT CRRT				
	No	17 (18.5%)	75 (81.5%)	92 (59.4%)
	Yes	20 (31.7%)	43 (68.3%)	63 (40.6%)
Intraoperative packed red blood cells (U)		3 (1 – 4)	2 (0 – 3)	2 (0 – 3)
Intraoperative fresh-frozen plasma (U)		4 (0 – 8)	0 (0 – 4)	0 (0 – 5)
Intraoperative cryoprecipitate transfusion				
	No	32 (24.1%)	101 (75.9%)	133 (85.8%)
	Yes	5 (22.7%)	17 (77.3%)	22 (14.2%)
Intraoperative platelet transfusion				
	No	21 (19.4%)	87 (80.6%)	108 (69.7%)
	Yes	16 (34.0%)	31 (66.0%)	47 (30.3%)
Length of ICU stay		11 (6 – 25)	6.5 (4 – 12)	7 (4 – 14)
Pre-OLT days of RRT				
	No	20 (18.3%)	89 (81.7%)	109 (70.3%)
	≤ 14 days	12 (32.4%)	25 (67.6%)	37 (23.9%)
	> 14 days	5 (55.6%)	4 (44.4%)	9 (5.8%)

Data are displayed as median (interquartile range - IQR) or as count (percentage). RRT, renal replacement therapy; OLT, Orthotopic liver transplantation; MELD, Model for End-Stage Liver Disease; Scr, serum creatinine; CRRT, continuous renal replacement therapy; HD, hemodialysis; ICU, intensive care unit.

*N = 149

**Fig 1:**
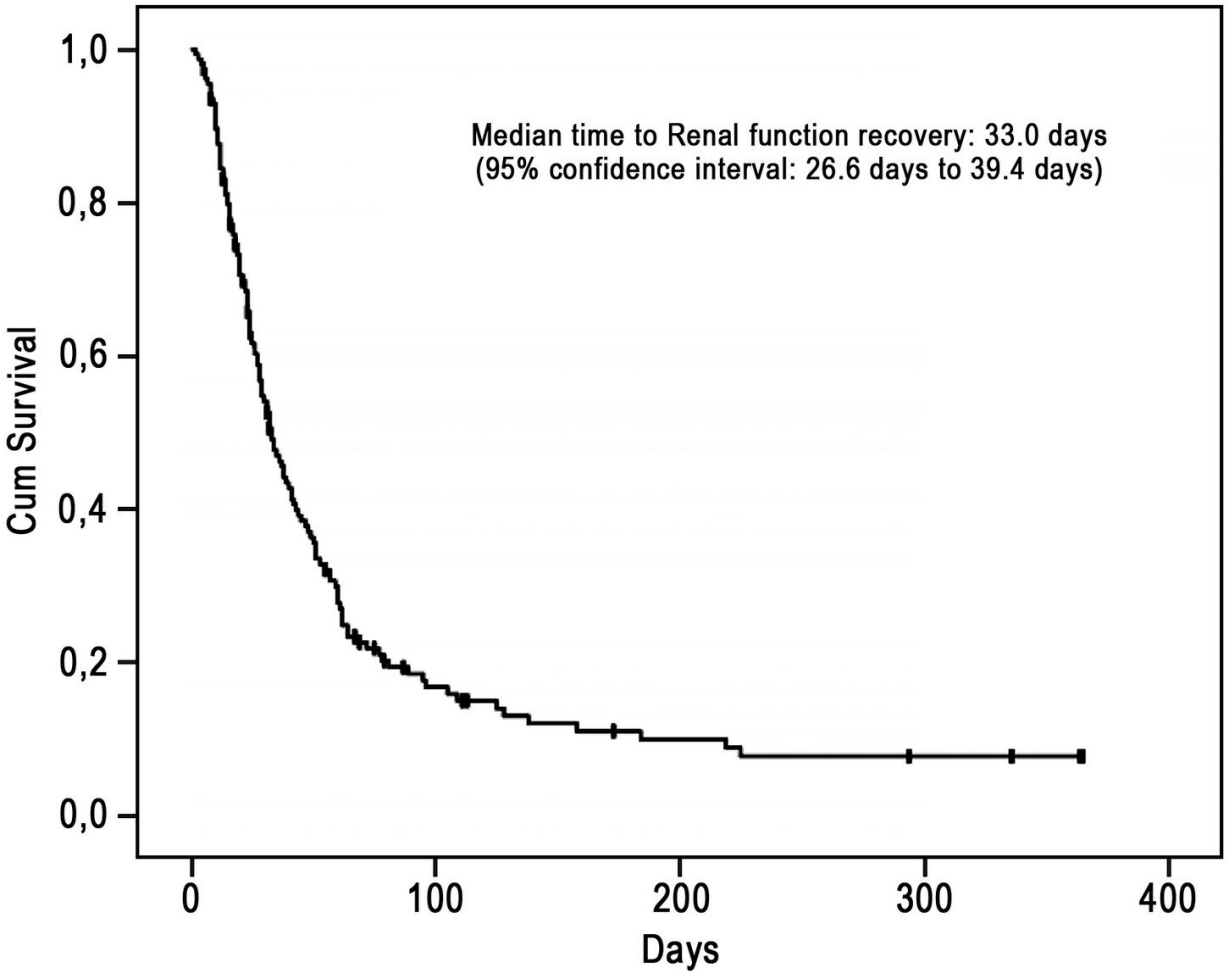
Time to renal function recovery.

At 90 days post-OLT, 118 (76%) patients had been removed from dialysis, and 16 (10%) patients had died while undergoing HD (Fig 1). At the one-year follow-up, a total of 129 (83%) patients had been removed from dialysis, 19 (12%) patients had died while undergoing HD, 2 (1%) patients had subsequently received a kidney transplant, and only 5 (3%) patients were currently undergoing HD. The median recovery time of the post-OLT patients was 33 (27–39) days. For the patients whose renal function recovered, 225 days was the longest duration of HD.

### Risk factors for renal function recovery

Univariate Cox regression analyses revealed that age; etiology of liver disease; pre-OLT hypertension status; Scr level > 1.5 mg/dL or undergoing RRT at the time of transplant; transfusion of intraoperative packed red blood cells, FFP and intraoperative platelets; undergoing pre-OLT intermittent HD and post-OLT CRRT; and the duration of pre-OLT RRT were important predictors of renal function recovery (Table 2). These variables were included in a multivariate model.

**Table 2:** Univariate Cox regression analysis of renal function recovery.

		Median time until renal function recovery	95% confidence interval		Log-rank p
			Lower	Upper	
General		33.0	26.6	39.4	
Gender	Female	32.0	26.3	37.7	0.632
	Male	34.0	22.9	45.1	
Age (years)	≤ 53.1	28.0	21.8	34.2	**0.106**
	> 53.1	43.0	27.2	58.8	
Etiology of liver disease	Alcoholic cirrhosis	32.0	27.9	36.1	**0.008**
	Fulminant hepatic failure	23.0	19.2	26.8	
	Other	41.0	26.0	56.0	
	Viral hepatitis	34.0	24.2	43.8	
Pre-OLT diabetes mellitus	No	31.0	25.3	36.7	0.354
	Yes	41.0	22.8	59.2	
Pre-OLT hypertension	No	32.0	27.3	36.7	**0.035**
	Yes	42.0	11.5	72.5	
Scr level > 1.5 mg/dL or in RRT at the time of listing	Scr ≤ 1.5 mg/dL	32.0	26.1	37.9	0.881
	Scr > 1.5 mg/dL	38.0	23.9	52.1	
	RRT	44.0	24.4	63.6	
MELD	≤ 27	32.0	24.0	40.0	0.712
	> 27	33.0	22.0	44.0	
Scr level > 1.5 mg/dL or undergoing RRT at the time of transplant	Scr ≤ 1.5 mg/dL	29.0	23.3	34.7	**0.049**
	Scr > 1.5 mg/dL	29.0	3.4	54.6	
	RRT	43.0	19.2	66.8	
Early re-OLT	No	32.0	21.5	42.5	0.183
	Yes	33.0	29.9	36.1	
Intraoperative packed red blood cells	No	23.0	18.1	27.9	**0.006**
	Yes	41.0	30.5	51.5	
Intraoperative fresh-frozen plasma	No	29.0	23.7	34.3	**0.005**
	Yes	40.0	25.6	54.4	
Intraoperative cryoprecipitate transfusion	No	32.0	25.7	38.3	0.659
	Yes	34.0	14.5	53.5	
Intraoperative platelet transfusion	No	29.0	22.6	35.4	**0.045**
	Yes	38.0	24.0	52.0	
Length of ICU stay	≤ 7 days	29.0	23.7	34.3	0.276
	> 7 days	37.0	30.3	43.7	
Hepatocellular carcinoma	No	33.0	25.3	40.7	0.734
	Yes	33.0	23.6	42.4	
Pre-OLT CRRT	No	32.0	26.7	37.3	0.384
	Yes	41.0	32.6	49.4	
Pre-OLT intermittent HD	No	31.0	25.6	36.4	**0.032**
	Yes	44.0	16.8	71.2	
Post-OLT CRRT	No	29.0	24.9	33.1	**0.096**
	Yes	41.0	28.1	53.9	
Pre-OLT days of RRT	No	31.0	26.2	35.8	**0.066**
	≤ 14 days	40.0	34.4	45.6	
	> 14 days *	--	--	--	

RRT, renal replacement therapy; OLT, orthotopic liver transplantation; MELD, Model for End-Stage Liver Disease; Scr, serum creatinine; CRRT, continuous renal replacement therapy; HD, hemodialysis; ICU, intensive care unit.

* Nine patients had > 14 days of pre-OLT RRT; of these, 4 recovered renal function, i.e., 55.5% were censored, which makes it impossible to estimate the median time to renal function recovery in this group.

In the multivariate analysis, fulminant hepatic failure as the cause of liver disease (HR = 3.39, 95% CI = 1.81 to 6.35, p < 0.001); the absence of pre-OLT hypertension (HR = 1.82, 95% CI = 1.12 to 2.96, p = 0.016); lower intraoperative FFP transfusion volume (HR = 0.94, 95% CI = 0.89 to 0.99, p = 0.019) and not undergoing pre-OLT intermittent HD (HR = 1.68, 95% CI = 1.05 to 2.7, p = 0.032) were associated with performing RRT for less than 90 days. Not undergoing post-OLT CRRT was also a marginally significant predictor of removal from dialysis (HR = 1.43, 95% CI = 0.95 to 2.14, p = 0.088; Table 3).

**Table 3:** Multivariate Cox regression analysis of renal function recovery.

		HR	95% confidence interval		P
			Lower	Upper	
Etiology of liver disease	Alcoholic cirrhosis	1.53	0.84	2.78	0.168
	Fulminant hepatic failure	3.39	1.81	6.35	<0.001
	Other	1.07	0.69	1.67	0.761
	Viral hepatitis (reference)	--	--	--	
Pre-OLT hypertension	Yes (reference)	--	--	--	
	No	1.82	1.12	2.96	0.016
Intraoperative cryoprecipitate transfusion		0.94	0.89	0.99	0.019
Pre-OLT intermittent HD	Yes (reference)	--	--	--	
	No	1.68	1.05	2.70	0.032
Post-OLT CRRT	Yes (reference)	--	--	--	
	No	1.43	0.95	2.14	0.088

OLT, orthotopic liver transplantation; HD, hemodialysis; CRRT, continuous renal replacement therapy.

## Discussion

Renal failure in cirrhotic patients is a challenging complication with a significant impact on mortality both before and after OLT [6, 7]. As the Scr level is a component of the MELD score, the number of patients with renal dysfunction who undergo OLT and the proportion of OLTs performed in combination with kidney transplants have increased [8, 9]. Patients with normal or mild impairment of renal function pre-OLT whose status deteriorates during the perioperative period may need RRT. A key point of this scenario is that it is important to determine the nature of renal dysfunction and anticipate whether kidney function could recover after post-OLT liver function recovery. In the present study, we evaluated 155 stable post-OLT patients who did not have chronic irreversible renal disease diagnosed preoperatively and required RRT (HD) post-intensive care hospitalization. Our objective was to describe the average time to renal recovery and identity the factors associated with remaining on RRT > 90 days after OLT among patients undergoing RRT. We observed that at 90 days post-OLT, 21 patients (14%) were undergoing RRT, and after 1 year, only 5 (3%) remained on dialysis. The proportion of patients in our study who recovered renal function was higher than that observed in a previous, larger study [4].

We did not aim to investigate the incidence of post-OLT RRT in our center, as we did not evaluate patients who required RRT in the intensive care unit environment post-OLT. Many of those critical patients underwent continuous dialysis modalities and might have had an increased risk of mortality. Our focus was on patients who were stable on dialysis and no longer needed intensive care but had an undetermined renal function prognosis. Age; etiology of liver disease; pre-OLT hypertension status; Scr level > 1.5 mg/dL or undergoing RRT at the time of transplant; transfusion of intraoperative packed red blood cells, FFP and intraoperative platelets; undergoing pre-OLT intermittent HD and post-OLT CRRT; and the duration of pre-OLT RRT were significant predictors of the need for RRT > 90 days after OLT in the univariate analysis. However, in the multivariate analysis, fulminant hepatic failure; the absence of pre-OLT hypertension; lower intraoperative FFP transfusion volume; and not undergoing pre-OLT intermittent HD were associated with performing RRT for less than 90 days.

A previous study found that the duration of renal dysfunction pre-OLT (defined as Scr level ≥ 1.5 mg/dL) was correlated with creatinine elevation 6 and 12 months following liver transplantation [10]. Similar results have been found among patients with kidney dysfunction for more than 12 weeks prior to OLT (pretransplant Scr level ≥ 2 mg/dL); in a previous study, these patients had increased risk of poor post-transplant renal outcomes (estimated glomerular filtration rate ≥ 20 mL/minute within 3 years post-transplant) [11]. Our data are in accordance with a study that evaluated 1041 liver transplant recipients who were on RRT at the time of OLT. In this study, 707 recipients (67.9%) had spontaneous recovery of renal function after liver transplantation, and those who recovered spontaneously had a significantly shorter course of RRT during the pre-transplant period (15.6 versus 36.6 days, p < 0.001) [4]. Similarly, Sharma et al. found that among surviving recipients who underwent acute RRT before OLT without subsequent kidney transplant, the majority recovered renal function within 6 months, and longer pre-OLT RRT duration was significantly associated with increased risk of renal nonrecovery (HR = 1.04 per day, 95% CI = 1.02 to 1.05, p < 0.001) [12].

Fulminant hepatic failure is defined as the rapid development of acute liver injury with severe impairment of synthetic function and hepatic encephalopathy in a patient without obvious previous liver disease [13]. AKI is common in patients with fulminant hepatic failure. Based on a retrospective analysis of data from 1604 patients with acute liver failure, Tujius et al. found that 70% developed AKI, 30% received RRT, and, in accordance with previous reports, outcomes were negatively affected by AKI [14, 15]. The need for RRT has also been identified as an independent prognostic factor of poor outcomes after OLT for fulminant hepatic failure [16]. Conversely, Leithead et al. did not find an association between perioperative AKI or RRT and chronic kidney disease in patients who received transplants due to acute liver failure [17]. They proposed that the failure of perioperative renal dysfunction to impact long-term posttransplant renal outcomes in patients with acute liver failure could reflect the duration of renal impairment as the duration of renal dysfunction appears to be a key determinant of chronic renal impairment in patients who receive transplants due to chronic liver disease [10, 17]. Nevertheless, it was observed that patients with fulminant hepatic failure and AKI rarely developed chronic kidney disease, which corroborates our finding that fulminant hepatic failure is a predictor of the need for RRT < 90 days after OLT [14].

Intraoperative blood transfusion is known to affect postoperative renal outcomes in OLT patients [18–20]. A biologically plausible explanation for this association is that kidney hypoperfusion secondary to intraoperative bleeding represents an additional renal insult in patients with pre-OLT AKI. In fact, Sirivatanauksorn et al. retrospectively observed that prolonged intraoperative hypotension and postoperative hypotension were independent risk factors for AKI after OLT [21]. Therefore, bleeding secondary to intraoperative coagulopathy could have contributed to our finding that the need for FFP transfusion was negatively associated with post-OLT recovery of renal function.

According to our data, pre-OLT hypertension was also associated with a higher probability of remaining on dialysis > 90 days post-OLT. Rueggeberg et al. observed that pretransplant hypertension was a risk factor for AKI after OLT [18]. Although we did not perform pre-OLT kidney biopsies to provide histological evidence of the degree of associated hypertensive nephrosclerosis in this subgroup of patients, it was not surprising that a classic risk factor for chronic kidney disease in the general population could also impact patients with subsequent AKI, as a similar association was previously observed in diabetic patients who underwent OLT [11, 12].

A previous study found that among patients who developed end-stage renal disease after OLT, only 27% who underwent HD were alive 6 years after the onset of renal failure; this finding is substantially lower than the 71.4% survival rate among those who subsequently received a kidney transplant [22]. Likewise, a Canadian registry study found significantly decreased survival in post-OLT patients who underwent RRT compared with matched nontransplant chronic dialysis control patients, with 5-year patient survival rates of 17% and 43% for the post-OLT patients and controls, respectively (p = 0.01) [23]. Thus, given the increasing frequency of renal transplant referrals for recipients of nonrenal solid-organ transplants [24], two decisions must be made: the appropriate time to list OLT patients undergoing RRT for a subsequent kidney transplant and whether they should be prioritized for kidney transplantation.

There is concern that recipients of OLT alone who undergo RRT may prematurely be considered part of the group of patients with end-stage renal disease who need subsequent kidney transplantation. This approach could compromise the pool of renal organs for those with end-stage renal disease alone. Percutaneous renal biopsy may be helpful for determining the therapeutic strategy in patients with liver function improvement and lower risk of bleeding. According to the RIFLE (Risk, Injury, Failure, Loss, and End-stage kidney disease) definition of AKI, end-stage renal disease is the complete loss of kidney function (i.e., need for RRT) for more than 3 months [25]. Based on this criterion, the renal transplant community is authorized to list postoperative OLT patients for kidney transplantation after 3 months of RRT. In fact, according to a previous report, within a year of transplant, a small percentage of OLT patients had already undergone subsequent kidney transplantation [26]. However, how long after liver transplantation should kidney transplantation be performed? Hepatorenal syndrome, which is thought to be reversible with OLT alone, may contribute to the requirement for long-term RRT post-transplant. A previous study indicated that among 2112 adult deceased-donor OLT recipients who received acute RRT for ≤ 90 days pre-OLT, a nonrecovery rate of only 8.9% was observed among those who survived 6 months following OLT [12]. In contrast, in a series of 130 patients with hepatorenal syndrome who underwent OLT, 7 (6%) were found to have developed irreversible kidney failure post-transplant, and 5 died within 1 year [27]. Based on these unfavorable results, the authors recommend a minimum 60-day waiting period prior to considering subsequent kidney transplantation, regardless of donor type [27]. Nevertheless, our data suggest a longer period may be appropriate before adding a patient to the kidney transplant list to assure opportune kidney allocation, and a minimum 3-month waiting period is suggested; this approach may be particularly appropriate for patients who have had a diagnosis of fulminant hepatic failure, had no pre-OLT hypertension, received a lower transfused volume of intraoperative FFP and did not undergo pre-OLT intermittent HD. Our data provide practical information for transplant doctors that allows them to stratify patients in terms of the factors predicting whether they will no longer need RRT.
